# Tau accumulation in the retina promotes early neuronal dysfunction and precedes brain pathology in a mouse model of Alzheimer’s disease

**DOI:** 10.1186/s13024-017-0199-3

**Published:** 2017-08-03

**Authors:** Marius Chiasseu, Luis Alarcon-Martinez, Nicolas Belforte, Heberto Quintero, Florence Dotigny, Laurie Destroismaisons, Christine Vande Velde, Fany Panayi, Caroline Louis, Adriana Di Polo

**Affiliations:** 1 Department of Neuroscience and Centre de recherche du centre hospitalier de l’Université de Montréal (CRCHUM), Université de Montréal, 900 Rue Saint-Denis, Tour Viger, Room R09.720, Montréal, QC H2X 0A9 Canada; 2 Institut de Recherches Servier, 78290 Croissy-sur-Seine, France

**Keywords:** Alzheimer’s disease, Retinal ganglion cell, Tau, Axonal transport, Neurodegeneration

## Abstract

**Background:**

Tau is an axon-enriched protein that binds to and stabilizes microtubules, and hence plays a crucial role in neuronal function. In Alzheimer’s disease (AD), pathological tau accumulation correlates with cognitive decline. Substantial visual deficits are found in individuals affected by AD including a preferential loss of retinal ganglion cells (RGCs), the neurons that convey visual information from the retina to the brain. At present, however, the mechanisms that underlie vision changes in these patients are poorly understood. Here, we asked whether tau plays a role in early retinal pathology and neuronal dysfunction in AD.

**Methods:**

Alterations in tau protein and gene expression, phosphorylation, and localization were investigated by western blots, qPCR, and immunohistochemistry in the retina and visual pathways of triple transgenic mice (3xTg) harboring mutations in the genes encoding presenilin 1 (*PS1*M146 V), amyloid precursor protein (*APP*Swe), and tau (*MAPT*P301L). Anterograde axonal transport was assessed by intraocular injection of the cholera toxin beta subunit followed by quantification of tracer accumulation in the contralateral superior colliculus. RGC survival was analyzed on whole-mounted retinas using cell-specific markers. Reduction of tau expression was achieved following intravitreal injection of targeted siRNA.

**Results:**

Our data demonstrate an age-related increase in endogenous retinal tau characterized by epitope-specific hypo- and hyper-phosphorylation in 3xTg mice. Retinal tau accumulation was observed as early as three months of age, prior to the reported onset of behavioral deficits, and preceded tau aggregation in the brain. Intriguingly, tau build up occurred in RGC soma and dendrites, while tau in RGC axons in the optic nerve was depleted. Tau phosphorylation changes and missorting correlated with substantial defects in anterograde axonal transport that preceded RGC death. Importantly, targeted siRNA-mediated knockdown of endogenous tau improved anterograde transport along RGC axons.

**Conclusions:**

Our study reveals profound tau pathology in the visual system leading to early retinal neuron damage in a mouse model of AD. Importantly, we show that tau accumulation promotes anterograde axonal transport impairment in vivo, and identify this response as an early feature of neuronal dysfunction that precedes cell death in the AD retina. These findings provide the first proof-of-concept that a global strategy to reduce tau accumulation is beneficial to improve axonal transport and mitigate functional deficits in AD and tauopathies.

## Background

Tau, a member of the microtubule-associated protein family, plays a crucial role in many neurodegenerative diseases including Alzheimer’s disease (AD), corticobasal dementia, frontotemporal lobar degeneration, progressive supranuclear palsy, and glaucoma [1–4]. These disorders share similar features including abnormal tau phosphorylation [5–7], protein aggregation [7, 8], neurofibrillary tangle formation [9, 10], and neurotoxicity [4, 11–13]. Tau dysfunction has been well described in AD, the principal cause of dementia worldwide [14, 15], and occurs several decades before the appearance of cognitive deficits [16, 17]. At present, little is known about the early sequence of events leading to tau pathology in AD, highlighting the need to elucidate the interplay of molecular and cellular changes during the pre-symptomatic stages of the disease.

The retina is an integral part of the central nervous system (CNS) and has long been considered a window to the brain. The signal produced by light-sensitive photoreceptors is transmitted to bipolar cells and then to retinal ganglion cells (RGCs), which send information via their axons in the optic nerve to visual centers in the brain [18]. As an integral part of the CNS, it is no surprise that the retina is affected by the same neurodegenerative processes that disturb brain function [19]. Indeed, visual deficits are common and significant in AD [20]. Impaired contrast sensitivity, reduced visual acuity and abnormal motion perception are found in AD, and these correlate tightly with the severity of cognitive and behavioral defects [21–31]. For example, 50% of AD patients presented with profound loss of pattern and spatial vision, including contrast sensitivity [32]. Approximately 50% of AD patients and 33% of individuals diagnosed with mild cognitive impairment have substantial visual motion perception deficits [33]. A study on individuals with AD-related senile dementia showed that 44% had important deficits in visual sensitivity measured by automated perimetry [34]. Morphological and additional functional impairments have also been described in the retina of AD individuals suffering from AD including preferential RGC loss and thinning of the retinal nerve fiber layer [35–38], abnormal electroretinogram response [39], and reduced blood flow [40, 41].

Similar to the brain, tau inclusions and amyloid beta (Aβ) deposition have been described in the retina of AD patients and in animal models of the disease [42–46]. Transgenic mice carrying the human P301S tau mutation contain tau aggregates in the retina [46], and display RGC functional deficits, increased susceptibility to excitotoxic damage, and altered neurotrophic factor signaling [47, 48]. We recently reported key pathological changes of endogenous tau in glaucoma, an optic neuropathy characterized by selective RGC death and the leading cause of irreversible blindness worldwide [4]. Ocular hypertension, a major risk factor in glaucoma, triggered substantial tau changes reminiscent of AD including abnormal phosphorylation, missorting, and neurotoxicity [4]. Collectively, these findings suggest an association between tau alterations and retinal dysfunction, notably linked to RGC damage.

At present, a detailed characterization of the biochemical changes and cellular distribution of endogenous retinal tau and its impact on RGC function and survival during the early pre-symptomatic and prodromal stages of AD is lacking. To address this, we utilized the triple transgenic (3xTg) line [13]. The rationale for the choice of this AD mouse model was three-fold. First, the presence of mutations in the genes encoding presenilin 1 (*PS1*), amyloid precursor protein (*APP*), and tau (*MAPT)* have been identified as causing familial AD (*PS1*M146V, APPSWE) or tauopathies including frontotemporal dementia and parkinsonism linked to chromosome 17 (*MAPT*P301L) [49]. Second, unlike other models, the 3xTg mice develop the two cardinal features of AD, namely accumulation of Aβ plaques and neurofibrillary tangles composed of tau, thus phenocopying critical pathological aspects of the disease [13]. Third, the 3xTg mice have been well-characterized regarding the appearance of brain lesions and cognitive deficits, thus providing a timeframe for the characterization of pathological changes in the visual system. Our data demonstrate that, as early as 3 months of age and prior to the onset of reported cognitive defects [50], abnormally phosphorylated tau accumulates in the retina of 3xTg mice preceding its aggregation in the brain. Tau accumulation was primarily observed in RGC soma, dendrites and intraretinal axons, while tau in optic nerve axons was markedly reduced. Importantly, tau phosphorylation and missorting resulted in striking defects in anterograde axonal transport and age-dependent RGC neurodegeneration. Our study identifies novel alterations of endogenous retinal tau protein and neuronal dysfunction in the early stages of AD, thus offering the possibility of exploiting tau to modulate disease susceptibility and onset.

## Methods

### Experimental animals

The 3xTg mice bearing the human mutations in the genes encoding presenilin 1 (*PS1*M146V), amyloid precursor protein (*APP*Swe), and tau (*MAPT*P301L) [51], tau knockout mice (strain Mapt-tm1[EGFP]Klt/J), and age-matched littermate wild-type controls were purchased from Jackson Laboratories (Bar Harbor, ME) and maintained in our animal facility. Experiments were performed using 3 or 6 month-old female mice because they exhibit a more severe disease phenotype than their age-matched male counterparts [52]. All animal procedures were approved by the Centre de Recherche du Centre Hospitalier de l’Université de Montréal (CRCHUM) Animal Care Committee (approved protocol #N14024ADPs), and followed the guidelines of the Canadian Council on Animal Care.

### Western blot analyses

Whole retinas, optic nerves or brains (frontal cortex and hippocampus) were isolated and homogenized in ice-cold lysis buffer: 50 mM Tris pH 7.4, 1 mM EDTA, 150 mM NaCl, 1% NP-40, 5 mM Na fluoride, 0.25% Na deoxycholate, and 2 mM NaVO_3_ supplemented with protease and phosphatase inhibitors. Protein homogenates were centrifuged at 18,000 *g* for 5 min, and the supernatants were removed and resedimented to yield soluble extracts. Samples in Laemmli buffer were boiled for 5 min, resolved in 4–15% SDS polyacrylamide gradient gels (Bio-Rad Life Science, Mississauga, ON), and transferred onto nitrocellulose membranes (Bio-Rad Life Science). Blots were incubated in blocking solution (10 mM Tris pH 8.0, 150 mM NaCl, 0.1% Tween-20 and 5% milk) for 1 h at room temperature, followed by overnight incubation at 4 °C with each of the following primary antibodies: total tau (K9JA, 1 μg/ml, Dako North America, Carpinteria, CA), phospho-tau S396-S404 (PHF1, 1:100, gift of P. Davies, Albert Einstein College of Medicine, Bronx, NY), phospho-tau S199 (PS199, 1 μg/ml, Invitrogen, Burlington, ON), phospho-tau S202-T205 (AT8, 0.8 μg/ml, Thermo-Fisher Scientific, Waltham, MA), MC-1 (1:100, gift of P. Davies), ALZ-50 (1:100, gift of P. Davies), or β-actin (0.5 μg/ml, Sigma-Aldrich, Oakville, ON). Membranes were washed and incubated in peroxidase-linked donkey anti-mouse or donkey anti-rabbit antibodies (0.5 μg/ml, GE Healthcare, Mississauga, ON). Blots were developed with a chemiluminescence reagent (ECL, Amersham Biosciences, Arlington Heights, IL) and exposed to X-OMAT imaging film (Eastman Kodak, Rochester, NY). Densitometry was performed on scanned autoradiographic films using the ImageJ software (http://imagej.nih.gov/ij/). Films were obtained from at least three independent western blots each carried out using retinal samples from different groups.

### Retina and optic nerve immunohistochemistry

Animals were perfused with 4% paraformaldehyde and the eyes and optic nerves were rapidly dissected. Tissue was embedded in optimal cutting temperature compound (Tissue-Tek, Miles Laboratories, Elkhart, IN), and retinal (16 μm) or optic nerve (12 μm) cryosections were collected onto Superfrost Plus microscope slides (Thermo-Fisher Scientific). The following primary antibodies were added to retinal or optic nerve sections in blocking solution and incubated overnight at 4 °C as described [53]: total tau (K9JA, 2 μg/ml, Dako), tubulin isoform βIII (TUJ1, 2.5 μg/ml; Sigma-Aldrich), or neurofilament H (NF-H, 20 μg/ml, Sternberger Monoclonals Inc., Lutherville, MA). For whole-mounted retinas, tissue was permeabilized overnight at 4 °C in blocking solution, rinsed and incubated for 5 days at 4 °C in the following primary antibodies: total tau (K9JA, 2 μg/ml, Dako), RNA-binding protein with multiple splicing (RBPMS, 1:1000, PhosphoSolutions, Aurora, CO), or NF-H (20 μg/ml, Sternberger Monoclonals Inc.). Sections or whole retinas were washed and incubated with secondary donkey anti-rabbit or anti-mouse Alexa Fluor 594 and 488 (2 μg/ml, Life Technologies, Eugene, OR). Fluorescent labeling was observed using a Zeiss Axio Observer (Carl Zeiss, Canada) or a Leica SP5 confocal microscope (Leica Microsystems Inc., Concord, ON). All retinal and optic nerve images were acquired under identical conditions using the same illumination intensity, time exposure, and magnification, with careful attention to avoid signal saturation and/or bleaching. The areas sampled were selected using an unbiased stereological sampling method as described (http://www.stereology.info).

### Reverse transcription and quantitative real time PCR (qPCR)

Total RNA was isolated from individual retinas using the RNEasy Mini kit (Qiagen Inc., Valencia, CA). cDNAs were generated from 1 μg of total RNA using the QuantiTect Reverse Transcription Kit (Qiagen Inc.). Real-time PCR was performed using TaqMan probes and primers that target exon 5, expressed by all tau isoforms (pan-tau, catalog # Rn01495715), exon 4a specific to big tau (catalog # Rn01495711), or β-actin RNA as control (catalog # 4331182) (Applied Biosystems, Waltham, MA). Amplification was performed using the 7900HT Fast Real-Time PCR System (Applied Biosystems) with the following cycle conditions: 95 °C for 15 s, 60 °C for 1 min, 72 °C for 1 min. Reactions were run in triplicates for each sample and the 2^-ΔΔ^Ct formula was used for the calculation of differential gene expression.

### Axonal transport measurement

Anterograde axonal transport was assessed by injection of cholera toxin β subunit (CTβ) conjugated to Alexa Fluor 488 (Molecular Probes, Life Technologies, Eugene OR) as described previously [54, 55]. CTβ is a reliable marker of active transport and has been consistently used to assess RGC anterograde transport to the superior colliculus [56, 57]. CTβ (1% diluted in sterile PBS, total volume: 1 μl) was injected intravitreally using a custom-made sharpened microneedle generated from a borosilicate glass capillary tube (5 μl, World Precision Instruments, Sarasota, FL) as previously described by us [58]. Briefly, the glass capillary was pulled using a two stage needle puller (PC-10, Narishige International, Amityville, NY) to produce thin microneedles of 6 cm in length. Under a dissecting microscope, a sharp blade was used to carefully create an opening at the tip of the microneedle. The resulting opening had an elliptical shape with a major and minor axis diameter of approximately 190 μm and 70 μm, respectively. The tip of the microneedle was then beveled using a micropipette bevelling system until the tip was sharp and the edges were flat and smooth [58]. For intravitreal injections, animals were anesthetized with isoflurane (2%, 0.8 L/min). The upper eyelid was gently retracted and the tip of the glass microneedle positioned at a 45° angle relative to the ocular surface. A light pressure was exerted to insert the microneedle through the conjunctiva, sclera and retina into the vitreous cavity. The tracer was injected and the needle was retracted slowly to avoid reflux. This route of administration avoided injury to ocular structures, including the iris and the lens. A small drop of antibiotic was applied topically after the surgery (Vigamox, 0.5%, Alcon Canada Inc., Mississauga, ON), and there were no signs of post-operative infection or inflammation. Animals were perfused transcardially with 4% paraformaldehyde three days after CTβ administration; brains were removed and embedded in optimal cutting temperature compound (Tissue-Tek, Miles Laboratories). Serial coronal cryosections of the entire superior colliculus from each animal were obtained using a cryostat (50-μm thickness). Seven sections per superior colliculus, from rostral to caudal, were selected using an unbiased stereological sampling method. Sections were photographed digitally using the Zeiss Axio Observer fluorescent microscope with Apotome (Carl Zeiss) and the area of the CTβ signal in each section was measured using the Imaris MeasurementPro module (Bitplane, South Windsor, CT). The total CTβ signal in each superior colliculus was calculated using the formula: total CTβ area = ΣCTβ section area/ssf x asf x tsf [59]. The section sampling fraction (ssf) was the number of sections analyzed over the total number of sections obtained from each superior colliculus (7/36), the area sampling fraction (asf) was the area sampled divided by the total area (1), and the thickness sampling fraction (tsf) was the section thickness sampled divided by the total section thickness (1). This analysis provided a representative value of CTβ-positive area which was then multiplied by the width of the entire superior colliculus to yield the total CTβ volume. To confirm CTβ uptake by RGCs, whole retinas were incubated overnight at 4 °C with goat IgG against the RGC-specific marker brain-specific homeobox/POU domain protein 3a (Brn3a, 0.27 μg/ml, Santa Cruz Biotechnology, Santa Cruz, CA) followed by secondary Alexa Fluor 594 anti-goat IgG (1 μg/ml; Jackson ImmunoResearch Laboratories, West Grove, PA). Retinas were rinsed, mounted, and the total number of CTβ-positive and Brn3a-positive neurons was quantified by independent random stereological sampling.

### Intravitreal injections of short interfering RNA (siRNA)

The following siRNA sequences against Tau (siTau) were purchased: i) 5′-CCUAGAAAUUCC AUGACGA-3′, ii) 5′-GGACAGGAAAUGACGAGAA-3′ iii) 5′-GCUGAUAGGCAGUUUAC AA-3′, iv) 5′-UGGGUGGGCUAGAUAGAUA-3′ (ON-TARGETplus mouse Mapt siRNA-SMART pool, GE Dharmacon, Lafayette, CO). The following control siRNA (siCtl) sequence was used: 5′-UGGUUUACAUGUUGUGUGA-3′ (ON-TARGETplus, non-targeting siRNA #2, GE Dharmacon). Each siRNA was injected into the vitreous chamber using a custom-made sharpened glass microneedle as described above (3.8 μg/μl, total volume: 2 μl).

### Quantification of RGC soma

Mice were subjected to transcardial perfusion with 4% paraformaldehyde and retinas were dissected out and fixed for an additional 15 min. Free-floating retinas were blocked overnight at 4 °C in 10% normal goat serum, 2% bovine serum albumin, 0.5% Triton X-100 in PBS, and incubated with the RGC-specific marker RBPMS (1:1000, PhosphoSolutions) for 5 days at 4 °C. Retinas were then incubated with Alexa 594-coupled secondary antibody (2 μg/ml, Life Technologies) for 4 h at room temperature, washed, mounted with the nerve fiber layer side up, and visualized with a Zeiss Axio Observer (Carl Zeiss). RBPMS-labeled RGCs were counted within three square areas at distances of 0.25, 0.625 and 1 mm from the optic disc in each of the four retinal quadrants for a total of twelve retinal areas as described by us [4].

### Statistical analyses

Data analysis and statistics were performed using GraphPad Instat software (GraphPad Software Inc., San Diego, CA) by a Student’s *t*-test as indicated in the legends.

## Results

### Tau protein accumulation in the retina precedes build up in the brain

**Fig. 1:**
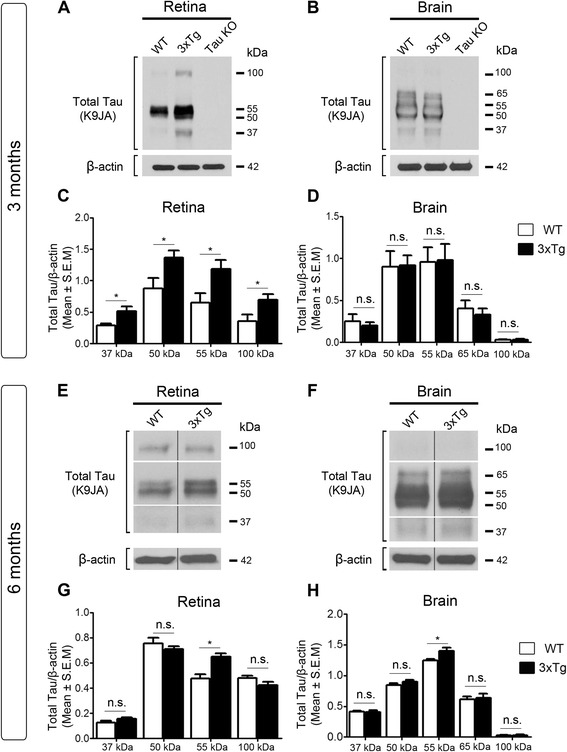
Tau protein accrues in the retina and precedes accumulation in the brain. **a** Representative western blots of soluble retinal extracts from 3-month-old 3xTg mice and wild-type (WT) age-matched controls probed with an antibody against total tau (K9JA) revealed the presence of four tau isoforms: 37-kDa, 50-kDa, 55-kDa and 100-kDa. All retinal tau isoforms were detected in both wild-type and 3xTg retinas. No signal was detected in tau knockout mice validating the specificity of the K9JA antibody. **b** Western blot analysis of brain homogenates from 3-month-old 3xTg mice and age-matched controls revealed four isoforms of 37-kDa, 50-kDa, 55-kDa and 65-kDa, while no signal was detected in brains from tau knockout mice. **c** Densitometry analysis showed a 1.8-, 1.6-, 2- and 2-fold increase in the 37-kDa, 50-kDa, 55-kDa, and 100-kDa tau variants, respectively, in retinal samples from 3xTg mice (*n* = 10) compared to WT controls (*n* = 8) (Student’s *t*-test, * = *p* < 0.05). **d** Quantitative analysis revealed no changes in brain tau levels between 3xTg mice (*n* = 10) and WT controls (*n* = 10) (Student’s *t*-test, n.s.: not significant, *p* > 0.05). **e**, **f** Western blots using retinal (**e**) or brain (**f**) samples from 6-month-old 3xTg mice demonstrated that only the 55-kDa tau form increased relative to age-matched WT controls. **g**, **h** Densitometry analysis indicated a ~ 1.4-fold increase in the 55-kDa tau isoform in the retina (**g**) and brain (**h**) of 3xTg mice compared to controls (3xTg: *n* = 5, WT: *n* = 4, Student’s *t*-test, * = *p* < 0.05). Vertical lines represent non-consecutive samples from the same gel

Visual deficits and pathological changes have been described in the retinas of AD patients [21, 31]. Therefore, we first asked whether the level of endogenous retinal tau was altered in 3xTg mice and, if so, whether it correlated with tau changes in the brain. For this purpose, retinal and brain protein samples from 3- and 6-month-old 3xTg mice were analyzed and compared to those from age-matched wild-type controls. These time points were selected because they precede the appearance of reported behavioral and cognitive defects in this model [50, 60]. Western blots of soluble retinal homogenates using an antibody against total tau (K9JA), which binds the microtubule binding domain of the protein irrespective of its phosphorylation state [61, 62], revealed the presence of four predominant tau isoforms of 37-kDa, 50-kDa, 55-kDa and 100-kDa (Fig. 1a). The 100-kDa band most likely corresponded to big tau, a high molecular weight tau isoform detected only in retinal and peripheral neurons [63–65]. Densitometry analysis showed that all four tau isoforms increased in 3-month-old 3xTg mouse retinas relative to age-matched wild-type controls (Fig. 1c). Analysis of cortical and hippocampal homogenates revealed the presence of four tau isoforms of 37-kDa 50-kDa, 55-kDa, and 60-kDa (Fig. 1b). In contrast to retina, however, the levels of all brain tau isoforms in 3-month-old 3xTg mice were similar to those in age-matched controls (Fig. 1d). No signal was detected in retinal or brain samples from tau null mice thus confirming the specificity of the K9JA antibody in these tissues (Fig. 1a, b). Western blot and densitometry analyses revealed an increase of the 55-kDa tau isoform in both retina and brain samples from of 6-month-old 3xTg mice, while all other isoforms remained unchanged (Fig. 1e-h). These results demonstrate that tau protein increases in transgenic retinas early in the disease process and precedes tau accumulation in the brain.

### Retinal tau undergoes epitope-specific and age-dependent phosphorylation changes in AD

**Fig. 2:**
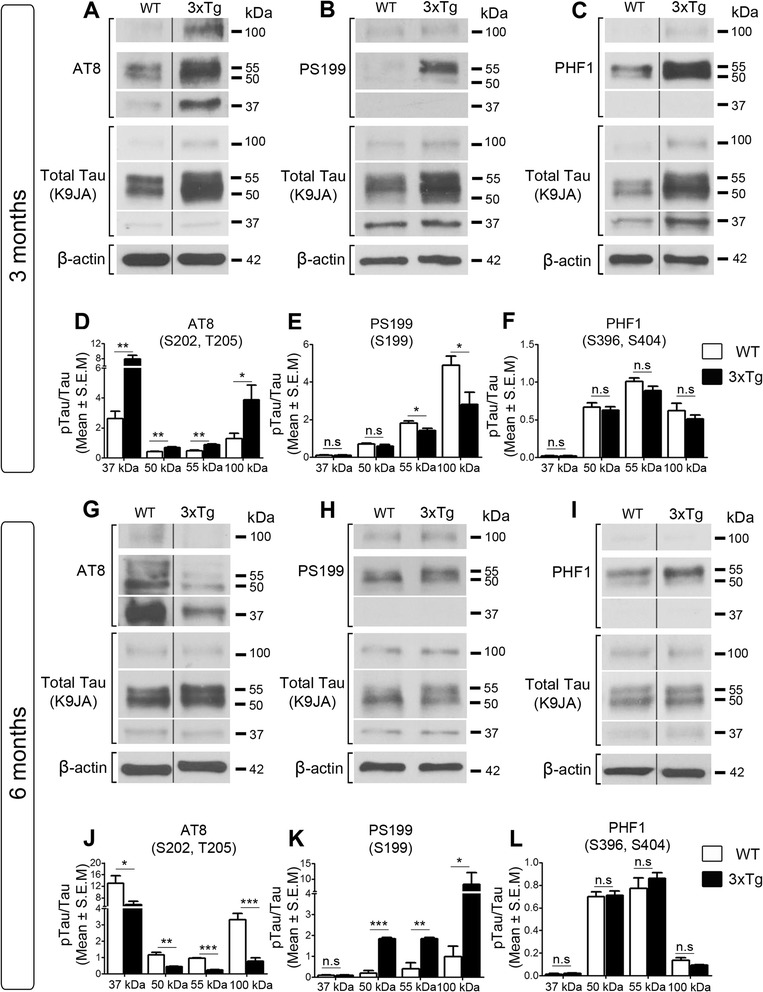
Retinal tau undergoes epitope-specific and age-dependent phosphorylation changes in AD**. a**-**c** Western blot analyses of retinal homogenates probed with the phospho-tau specific antibodies AT8, PS199, and PHF1 revealed alterations in tau phosphorylation in 3xTg relative to wild-type (WT) mice. **d**-**f** Densitometry analysis of phospho-tau relative to total tau revealed increased tau phosphorylation on S202/T205 (AT8) and reduced phospho-S199 (PS199) in 3xTg retinas relative to controls, while no change was detected on S396/S404 (PHF1) (3xTg: *n* = 5, WT: *n* = 4, Student’s *t*-test, * = *p* < 0.05, ** = *p* < 0.01). **g-i** Western blot analysis of tau phosphorylation in 6-month-old 3xTg retinas showed alterations on epitopes AT8 and PS199, but not PHF1, relative to controls. **j-l** Quantitative analysis confirmed decreased tau phosphorylation on S202/T205, increased phosphorylation on S199, and no change on PHF1 (3xTg: *n* = 5, WT: *n* = 4, Student’s *t*-test, * = *p* < 0.05, ** = *p* < 0.01, *** = *p* < 0.001, n.s.: not significant *p* > 0.05). Vertical lines represent non-consecutive samples from the same gel

Alterations in tau phosphorylation contribute to tau dysfunction in pathological conditions [14, 66, 67]. Therefore, we analyzed tau phosphorylation in retinal samples from 3xTg mice using the phospho-tau specific antibodies AT8 (S202, T205) [68, 69], PS199 (S199) [70], and PHF1 (S396, S404) [71, 72]. These antibodies were selected because they recognize phospho-specific epitopes that correlate with AD severity [73]. Visualization of western blots probed with AT8 or PHF1 revealed an increase in tau phosphorylation at these epitopes in 3-month-old 3xTg retinas (Fig. 2a, b). Densitometry analysis of phospho-tau signals with respect to total tau, which markedly increased at this age, confirmed greater tau phosphorylation on residues S202 and T205 of all isoforms (AT8, Fig. 2a, d), while a relative decrease at residue S199 was observed (PS199: 55-kDa, 100-kDa) (Fig. 2b, e). Conversely, 6-month-old 3xTg retinas displayed reduced phosphorylation with AT8 on all isoforms (Fig. 2g, j), and increased phosphorylation with PS199 (50-kDa, 55-kDa, 100-kDa) (Fig. 2h, k). No significant changes were detected with PHF1 in 3- or 6-month-old 3xTg retinas relative to controls (Fig. 2c–l).

**Fig. 3:**
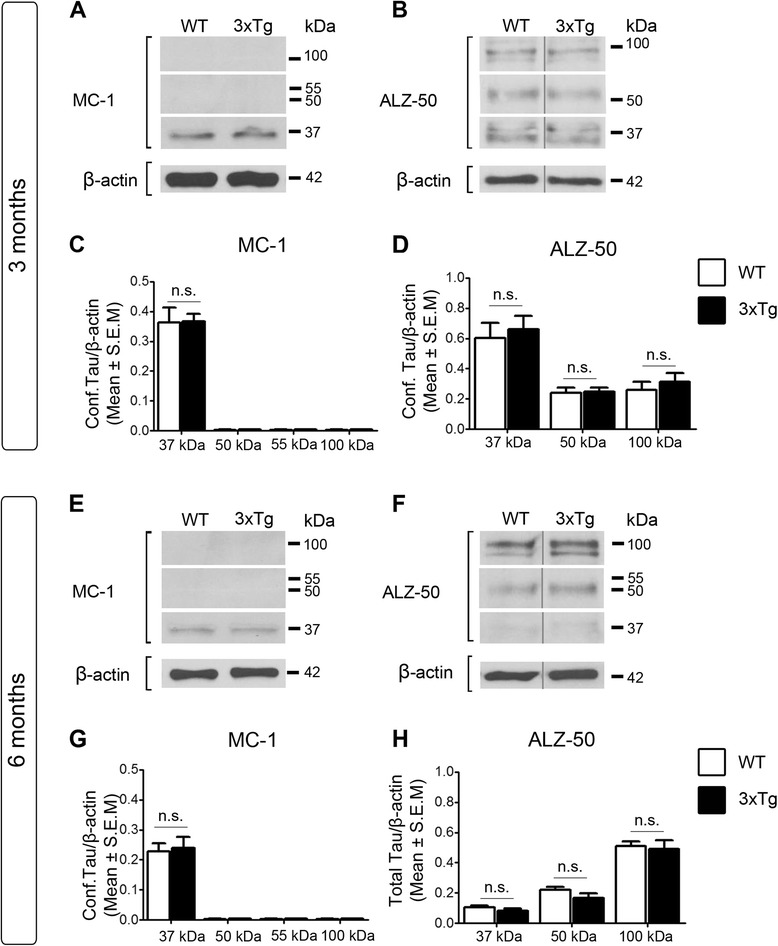
Lack of conformational tau changes in AD retinas. **a**, **b** Western blot analysis of retinal extracts from 3-month-old 3xTg mice probed with MC-1 or ALZ-50 antibodies demonstrated lack of changes in transgenic mice relative to controls. **c**, **d** Densitometry confirmed the lack of variations in tau conformation-dependent markers (3-month-old 3xTg: *n* = 5, 3-month-old WT: *n* = 4). **e-h** Western blots of retinal homogenates and densitometry analyses from 6-month-old mice also revealed absence of tau conformational changes between transgenic and control mice (6-month-old 3xTg: *n* = 5, 6-month-old WT: *n* = 4, Students *t*-test, n.s.: not significant *p* > 0.05). Vertical lines represent non-consecutive samples from the same gel

Phosphorylation can lead to changes in the conformation of tau protein during the course of AD, which can play a key role in pathological tau accumulation and cleavage [74, 75]. The conformation of tau in 3xTg retinas was investigated using the antibodies MC-1 and ALZ-50, which recognize the early folding back of the N-terminus on the microtubule domain in a hairclip configuration linked with tau aggregation [74, 76, 77]. No changes in conformation-dependent epitopes recognized by MC-1 or ALZ-50 were detected in 3- or 6-month-old 3xTg retinas relative to age-matched controls (Fig. 3). Together, these data demonstrate increased tau phosphorylation at AT8 epitopes in the early presymptomatic stages, whereas increased phosphorylation at PS199 appears at a later phase of the disease, in the absence of conformational changes. We conclude that retinal tau undergoes complex age-related and epitope-specific changes in AD.

### Tau accumulates in the RGC somatodendritic compartment and intraretinal axons

**Fig. 4:**
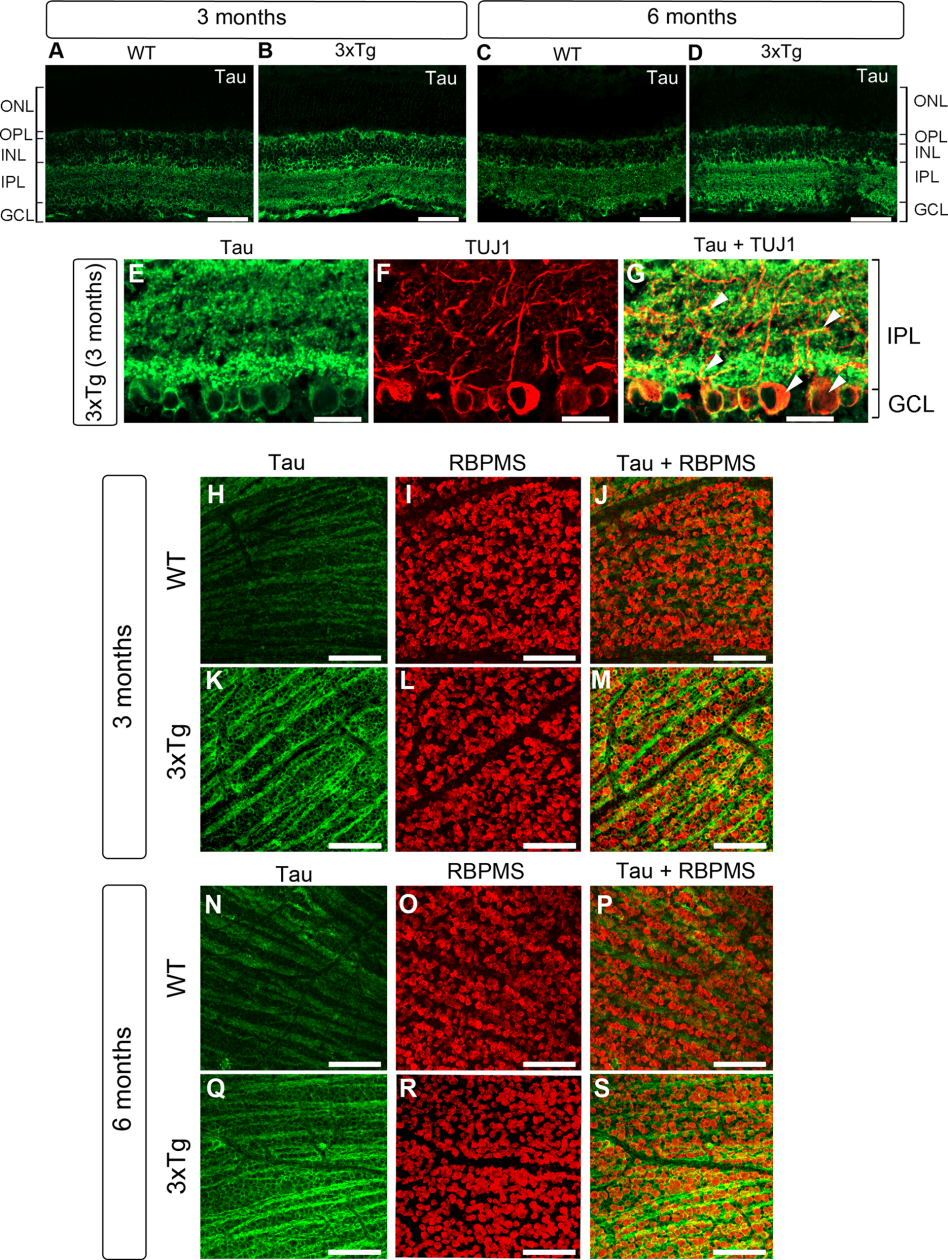
Tau accumulates in the somatodendritic compartment of RGCs. **a**-**d** Retinal immunohistochemistry using antibodies against total tau (K9JA) revealed marked tau upregulation in 3- and 6-month-old 3xTg retinas relative to age-matched wild-type (WT) controls. **e**-**g** Co-staining of tau with TUJ1, an RGC-specific marker, demonstrated tau accumulation in 3xTg RGC dendrites and somata (arrowheads) (*n* = 5/group). **h**-**s** Whole-mount retinal preparations from 3- and 6-month-old mice show co-localization of tau and RBPMS, a selective marker of RGC soma, demonstrating age-dependent accumulation of tau in RGC soma (*n* = 5/group). Scale bars: A–D = 50 μm, E-G = 25 μm, H-S = 50 μm. ONL: outer nuclear layer, OPL: outer plexiform layer, INL: inner nuclear layer, IPL: inner plexiform layer, GCL: ganglion cell layer

The cellular distribution of tau in the retina was investigated by immunohistochemistry using the K9JA antibody against total tau. In agreement with previous reports [4, 78], a low basal level of tau expression was found in all retinal layers except the outer nuclear layer (Fig. 4a, c). Consistent with our biochemical findings, retinal tau increased in 3-and 6-month-old 3xTg mice and its accumulation was more pronounced in the inner plexiform layer (IPL), where RGC dendrites are located (Fig. 4b, d). Labeling with an antibody against tubulin isoform βIII (TUJ1), an RGC-specific marker that strongly labels the soma and dendrites of these neurons [79, 80], confirmed localization of tau within the somatodendritic compartment (Fig. 4e-g). Further analysis of tau distribution on flat-mounted retinas using RBPMS, which selectively labels RGC soma [81, 82], confirmed tau accumulation in RGC bodies in 3xTg retinas relative to controls (Fig. 4h-m). A similar distribution of tau was observed in 6-month-old transgenic mice, with more robust tau build up at this age (Fig. 4n-s).

**Fig. 5:**
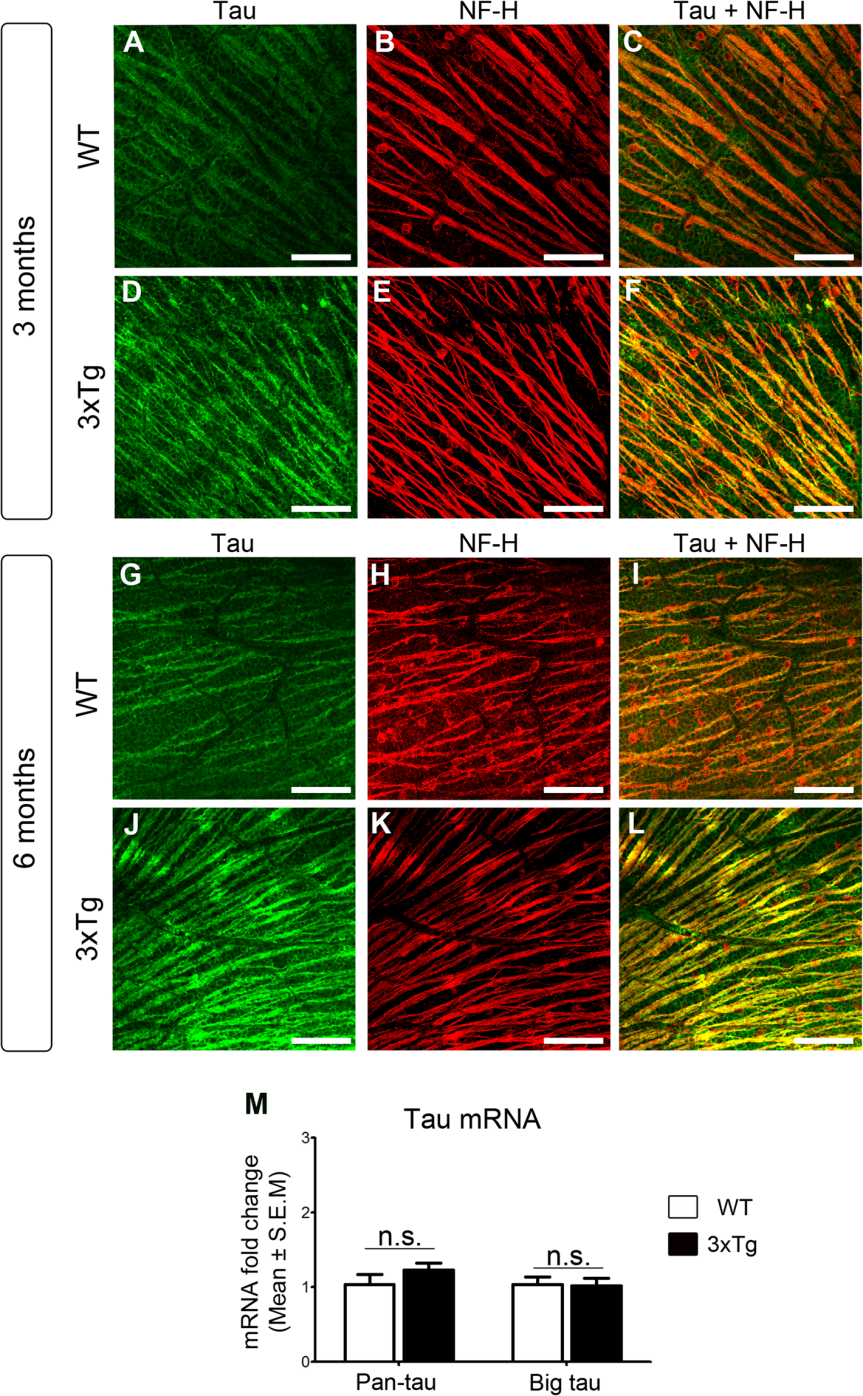
Tau accumulates in RGC intraretinal axons. **a**-**f** Whole-mounted retinas labeled with tau (K9JA) and the axonal marker NF-H revealed marked accumulation of tau in intraretinal axons of young (3 months) transgenic mice relative to controls. **g**-**l** Tau build up in intraretinal axons, visualized with NF-H, was more pronounced at 6 months of age. **m** Real-time qPCR analysis demonstrated no significant change in retinal tau gene expression (3xTg: *n* = 7, WT: *n* = 7, Student’s *t*-test, n.s.: not significant *p* > 0.05). Scale bars: **a**–**l** = 50 μm

Further analysis of tau expression in flat-mounted retinas, specifically changes in the intraretinal RGC axons, was investigated by co-localization of tau with the axonal marker neurofilament-H (NF-H). In wild-type retinas, low basal levels of tau protein were detected in RGC intraretinal axons, visualized with NF-H (Fig. 5a-c, g-i). In contrast, a pronounced increase of tau signal in NF-H-positive axons was detected in 3xTg retinas at both 3 and 6 months of age (Fig. 5d-f, j-l). To establish whether tau accumulation in the retina resulted from increased gene expression, real-time qPCR was performed using primers that recognized all tau isoforms (pan-tau) or big tau [63–65]. No significant changes in tau mRNA levels were detected in AD retinas compared to controls (Fig. 5m). These data indicate that tau accrues early in the AD retina, predominantly in RGC dendrites and intraretinal axons, and that this accumulation is not the result of increased gene expression.

### Tau is depleted from RGC axons in 3xTg optic nerves

**Fig. 6:**
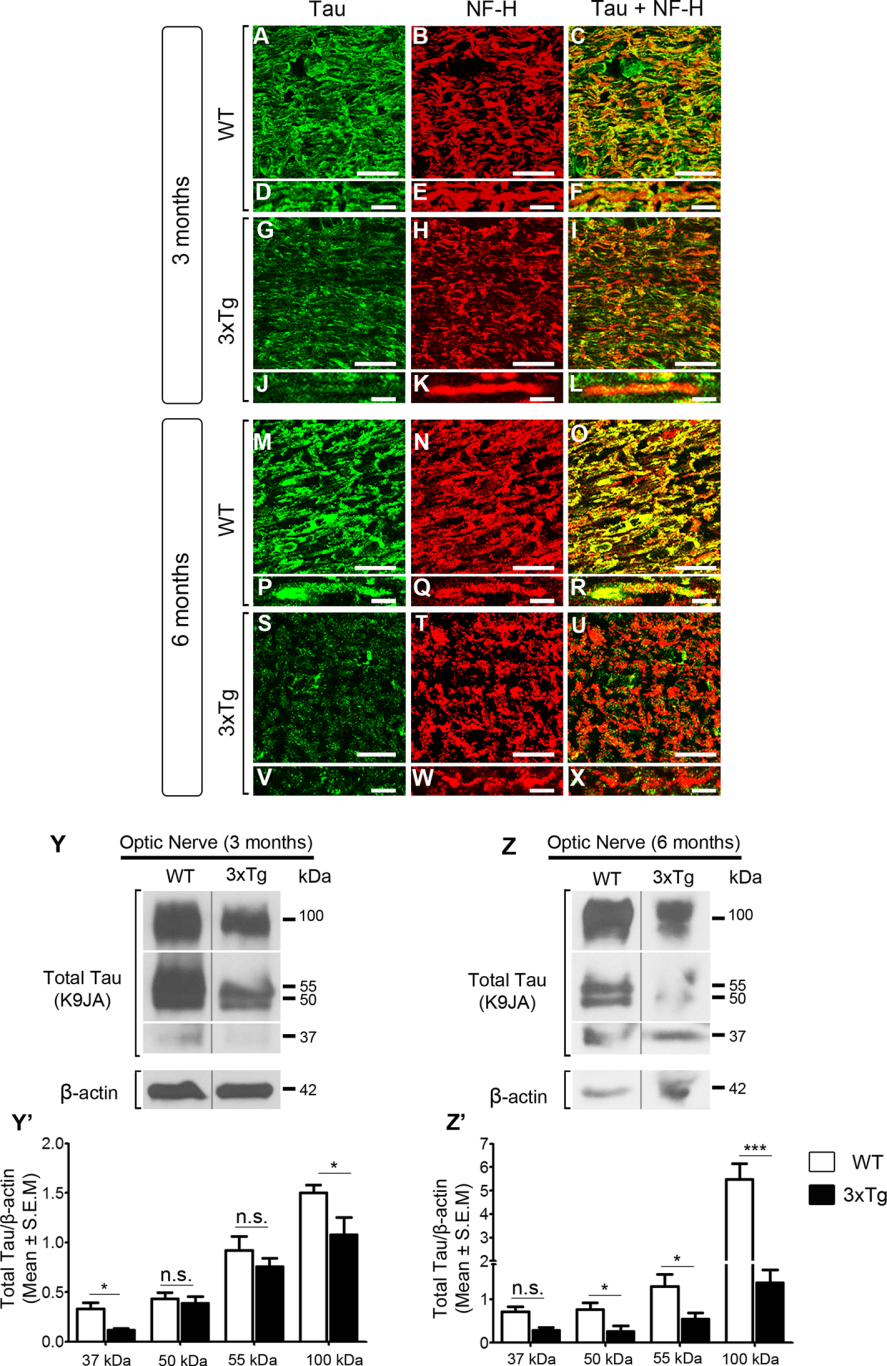
Tau is depleted from RGC axons in transgenic optic nerves. **a**-**c** Optic nerve cross sections colabeled with tau and NF-H display high levels of tau in RGC axons in 3-month-old wild-type (WT) mice. **d**-**f** Higher magnification images demonstrate co-localization of tau in NF-H-positive RGC axons. **g**-**l** In contrast, optic nerves from age-matched 3xTg animals show marked reduction of tau, which was not due to axonal loss as co-staining with NF-H confirmed an abundance of RGC axons. **m**-**x** Tau expression in 3xTg optic nerve axons was much reduced in older mice (6 months) as demonstrated by the marked loss of tau labeling in the optic nerve sections despite robust NF-H staining (*n* = 5/group). Scale bars: **a**–**c**, **g**-**i**, **m**-**o**, **s**-**u** = 10 μm (1000× magnification); **d-f**, **j-l**, **p-r**, **v**-**x** = 4 μm (2000× magnification). **y**, **z** Western blot analysis of tau expression in optic nerve protein homogenates from 3- and 6-month-old mice confirmed the loss of axonal tau in transgenic animals relative to controls (3-month-old 3xTg: *n* = 6, 3-month-old WT: *n* = 9, 6-month-old 3xTg: *n* = 4, 6-month-old WT: *n* = 4, Student’s *t* test, * = *p* < 0.05, n.s.: not significant *p* > 0.05). Vertical lines represent non-consecutive samples from the same gel

In normal physiological conditions, tau is enriched in axons with low levels found in dendrites and soma [83]. In AD and other tauopathies, tau detaches from axonal microtubules and accumulates in the somatodendritic compartment of affected neurons [84]. To assess whether the distribution of tau in RGC axons within the optic nerve was altered in 3xTg mice, we carried out confocal imaging of nerve cross sections colabeled with tau and the axonal marker NF-H. In control optic nerves, tau was enriched in RGC axons visualized with NF-H (Fig. 6a-c, m- o). In contrast, 3xTg optic nerves from both 3- and 6-month-old mice displayed a striking reduction in axonal tau protein (Fig. 6g-i, s-u). High magnification confocal images of individual optic nerve fascicles revealed that many NF-H positive axons were still detected in transgenic animals in spite of marked reduction of tau protein levels (Fig. 6j-l, v-x), indicating that a decrease in tau was not the result of axonal degeneration. Western blot analysis of optic nerve homogenates confirmed a visible reduction of tau protein in 3- and 6-month-old 3xTg mice (Fig. 6y, Y’, z, Z’). Taken together, our results demonstrate that tau is markedly reduced in RGC axons within the optic nerve early in the course of the disease.

### Anterograde transport impairment in 3xTg RGCs precedes neuronal death

**Fig. 7:**
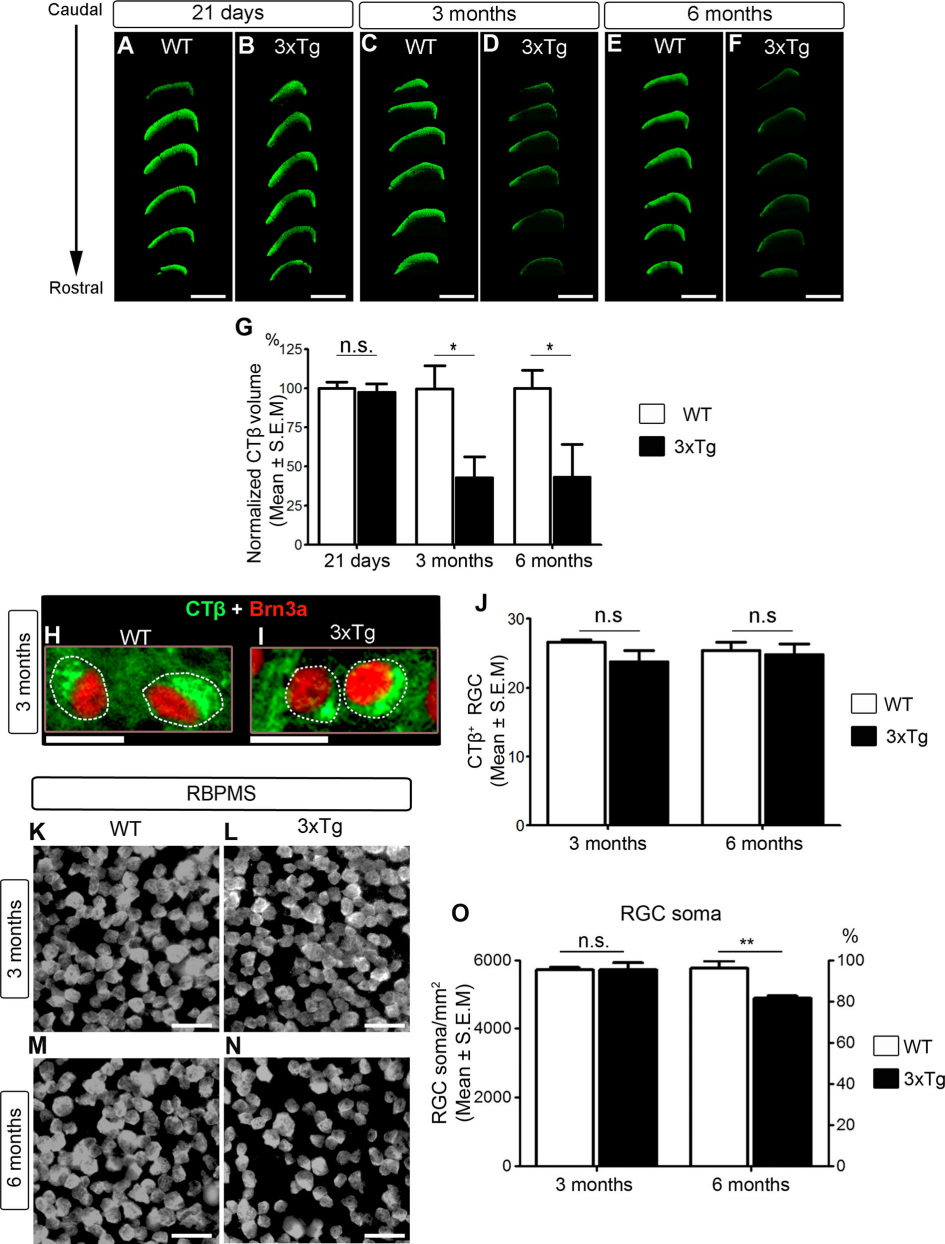
Anterograde transport impairment in 3xTg RGCs precedes neuronal death. **a**-**f** Unbiased stereological rostral to caudal sampling of the superior colliculus after CTβ injection showed no changes in CTβ labeling in 21-day-old wild-type or 3xTg mice. In contrast, a marked reduction of CTβ labeling was observed in both 3- and 6-month-old 3xTg mice relative to controls. **g** Quantification of the total CTβ-positive region in the superior colliculus demonstrated a striking loss of anterograde transport in transgenic mice compared to age-matched wild-type (WT) controls (3-month-old 3xTg: *n* = 5, 3-month-old WT: *n* = 3, 6-month-old 3xTg: *n* = 5, 6-month-old WT: *n* = 5, Student’s *t*-test, * = *p* < 0.05). **h**, **i** Co-labeling of CTβ (green) with the RGC-specific marker Brn3a (red) confirmed effective CTβ uptake by RGCs in both 3xTg and WT retinas. **j** Quantitative analysis confirmed that there was no difference in the number of CTβ- and Brn3a-positive RGCs between 3xTg and WT retina at 3 or 6 months of age (3-month-old 3xTg: *n* = 3, 3-month-old WT: *n* = 3, 6-month-old 3xTg: *n* = 3, 6-month-old WT: *n* = 4). **k-m** Flat-mounted retinas labeled with the RGC-specific marker RBPMS were used to quantify RGC density (survival). **o** Quantitative analysis of RGC density demonstrated absence of cell death in 3-month-old 3xTg mice, while only a modest loss was observed in 6-month-old transgenic animals relative to age-matched WT controls (3-month-old 3xTg: *n* = 5, 3-month-old WT: *n* = 6, 6-month-old 3xTg: *n* = 5, 6-month-old WT: *n* = 5, Student’s *t*-test, *p < 0.05, n.s.: not significant *p* > 0.05). Scale bars: A-D = 500 μm, F-G = 25 μm, K-*N* = 7.5 μm

RGCs, like other long projecting neurons, rely heavily on axonal transport for proper function. Anterograde transport impairment is recognized as an early sign of RGC damage and dysfunction [55]. Therefore, we examined whether the ability of RGCs to transport the anterograde tracer CTβ to terminals in the superior colliculus, the primary target of RGCs in the rodent brain [85–87], was altered in 3xTg mice. CTβ is an excellent reagent to study axonal transport because of its high sensitivity, ability to effectively move anterogradely or retrogradely from the injection site, dependency on intact microtubules thus serving as readout of active transport, capacity to label the entire neuron including extremely fine terminals, restriction from labeling fibers of passage, and demonstrated efficacy to label axonal tracts in the visual system following ocular administration [54–56, 88–96]. Alexa Fluor 488-conjugated CTβ was injected intravitreally and its accumulation in the contralateral superior colliculus was quantified using unbiased stereological sampling [54]. Mutant APP, PS1 and tau proteins are expressed throughout development in 3xTg mice, thus we first aimed to establish whether there were developmental alterations in axonal transport in young mice (21 days), a week after eye opening. Our data demonstrate that there was no difference in the amount of brain CTβ between wild-type and 3xTg at 21 days of age (Fig. 7a, b). In contrast, a substantial reduction in the CTβ-labeled volume was observed in 3- and 6-month-old 3xTg mice relative to age-matched controls (Fig. 7c-f). Quantification of total CTβ volume confirmed a 57% reduction in the superior colliculi of 3xTg mice suggesting major deficits in the ability of RGCs to transport cargos to their targets (Fig. 7g). These findings indicate that deficits in anterograde axonal transport in 3xTg mice are not of developmental origin, but rather reflect pathological changes detected early in the course of the disease.

To rule out that anterograde transport deficits were caused by the inability of 3xTg RGCs to uptake CTβ, we examined CTβ-injected retinas with Brn3a, a selective marker of RGC nuclei [97]. Abundant cytoplasmic CTβ within RGCs was observed at 24 h after tracer injection in both wild-type and transgenic retinas (Fig. 7h, i). Quantification of Brn3a-positive RGCs containing CTβ revealed a similar number of neurons in both 3xTg and control mice (Fig. 7j), thus confirming effective tracer uptake. To determine whether anterograde transport deficits reflected neuronal death, RGC density was quantified in 3- and 6-month-old transgenic retinas using the cell-specific marker RBPMS. A similar RGC density was found in 3xTg and wild-type mice at 3 months of age, confirming the absence of significant cell death at a time when major transport deficits are already apparent (Fig. 7k–o). In 6-month-old transgenic retinas, only a modest reduction in RGC density was detected relative to controls (~15%, Fig. 7m–o), therefore the substantial axonal transport loss at this age cannot be ascribed solely to retinal neuron death. Collectively, these results demonstrate that major deficits in axonal transport along RGC axons are a relatively early feature of neuronal dysfunction in AD pathology that precedes cell death.

### Selective tau knockdown improves RGC axonal transport

**Fig. 8:**
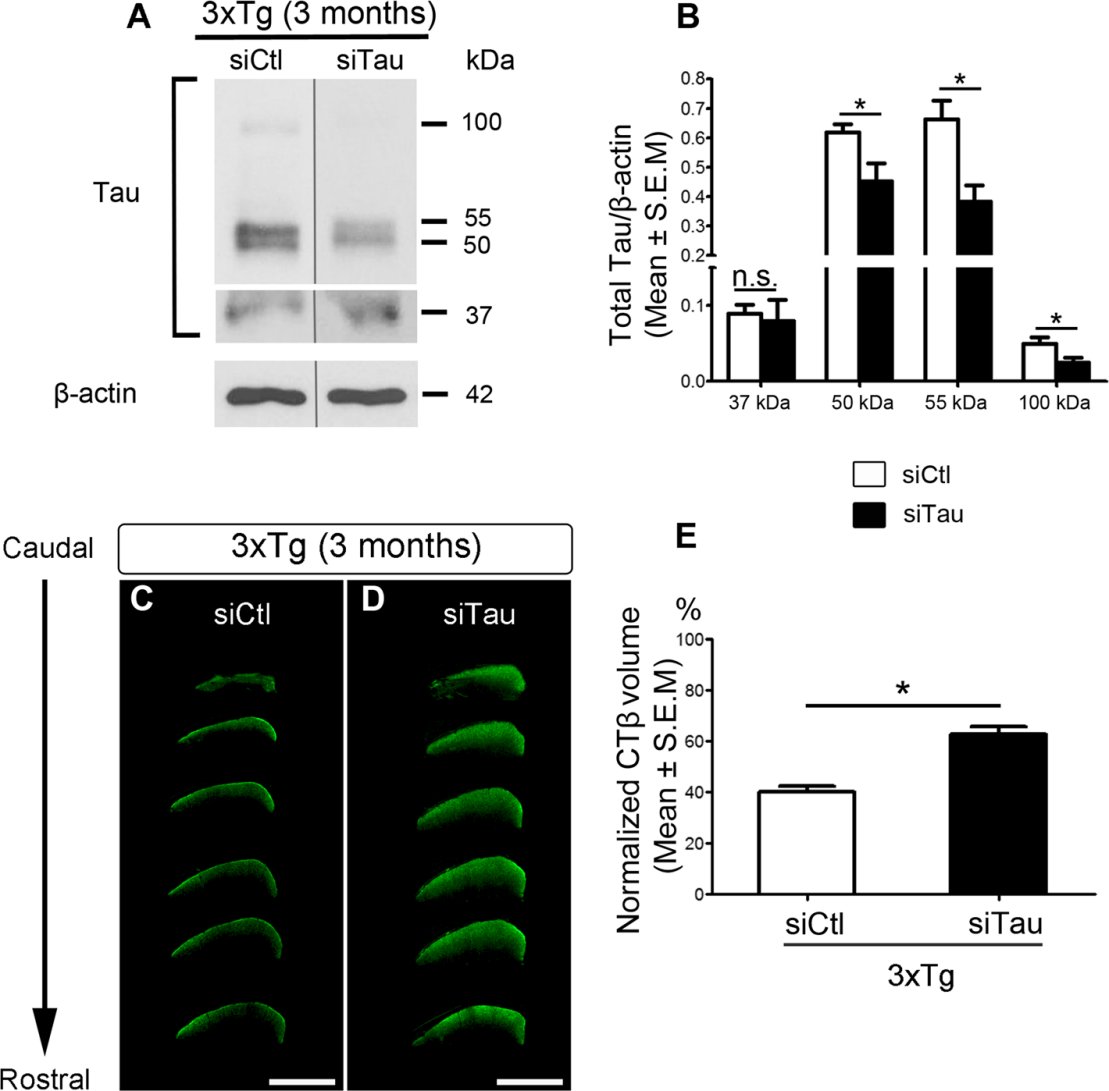
siRNA-mediated tau deletion improves axonal transport. **a**, **b** Western blot analysis of retinal homogenates from transgenic eyes treated with short interference RNA (siRNA). Eyes injected with siRNA against tau (siTau) showed a significant reduction in tau protein (50-kDa, 55 k-Da, and 100 k-Da) while a control siRNA (siCtl) had no effect (siCtl: *n* = 3; siTau: *n* = 3; Student’s *t*-test, * = *p* < 0.05). **c**, **d** Unbiased rostrocaudal stereological sampling of the superior colliculus after tracer injection shows increased CTβ in the brains of 3xTg mice that received siTau relative to siCtl-treated control mice. **e** Quantitative analysis of the total CTβ volume shows a significant increase of anterograde transport (20%) in siTau-treated mice compared to siCtl-treated controls (siTau: *n* = 5, siCtl: *n* = 6, Student’s *t*-test, * = *p* < 0.05). Scale bars: C, D = 500 μm

To investigate whether tau accumulation underlies the axonal transport deficits in 3xTg RGCs, we sought to decrease tau protein levels using siRNA followed by analysis of CTβ transport. First, we assessed the ability of a targeted siRNA against tau (siTau) to reduce retinal tau protein levels. We previously demonstrated that siRNA delivered by intravitreal injection is rapidly taken up by RGCs [98]. Western blot analysis of retinal extracts from 3-month-old 3xTg eyes that received siTau showed a significant reduction in tau protein relative to age-matched transgenic mice that received a control non-targeting siRNA (siCtl) (Fig. 8a). Quantitative analysis confirmed that siTau induced a 27%, 42% and 50% reduction of the 50-kDa, 55-kDa and 100-kDa tau isoforms, respectively, relative to control siRNA-treated eyes (Fig. 8b). Next, we investigated whether siRNA-mediated tau knockdown improved RGC axonal transport. For this purpose, siTau was injected intraocularly once a week for a total of three weeks. The multiple injection regimen was selected based on our previous findings that siRNA-mediated protein knockdown in the retina is transient [98]. Four days after the last siTau injection, CTβ was administered in the eye and the amount of the tracer in the superior colliculus quantified three days later. Visualization of caudal-to-rostral sections from the superior colliculus of siTau-treated 3xTg mice showed a marked increase in CTβ relative to transgenic mice that received control siCtl (Fig. 8c, d). Quantitative analysis confirmed that tau knockdown promoted a significant increase in anterograde axonal transport compared to control animals (20%, Fig. 8e). Our results demonstrate that attenuation of retinal tau levels improves axonal transport, suggesting that early tau accumulation in the retina impairs axonal function in AD.

## Discussion

Data presented here in a well-characterized mouse model of AD reveal profound alterations in tau within the retina and the visual pathways leading to neuronal dysfunction in vivo. First, we demonstrate that retinal tau accumulation in 3xTg mice occurs early and precedes pathological changes in the brain. Second, our data show that retinal tau undergoes age-related and epitope-specific changes in phosphorylation, which are independent of conformational modifications. Third, we found that tau build up occurs in the somatodendritic compartment and intraretinal axons of RGCs, whereas tau is depleted from optic nerve axons. Lastly, our results demonstrate that tau accumulation leads to substantial deficits in anterograde transport along 3xTg RGC axons, and that tau knockdown improves axonal transport. Collectively, this study reveals early and profound alterations in retinal tau leading to axonal dysfunction suggesting a role for pathological tau in visual deficits associated with AD.

Accumulation of pathological tau is a hallmark of AD and other tauopathies [1, 66, 99, 100]. Our data using the 3xTg mouse model, demonstrate early accumulation of tau in the retina prior to the onset of reported cognitive defects [50]. This finding is consistent with previous studies demonstrating increased retinal tau levels in murine models of tauopathies [45, 46, 48, 78]. The increase in retinal tau reported here was considerably more pronounced in younger mice than in older individuals. The expression of mutant tau in 3xTg mice is under the control of the Thy1 promoter [51], and Thy1 transcriptional activity has been shown to remain constant in the adult retina [81]. Therefore, it is unlikely that the marked age-dependent increase in retinal tau reported here is due to changes in Thy1 promoter activity. This is further supported by our finding that tau protein upregulation is not the result of increased gene expression. Instead, our results reveal a robust retinal response early in the course of the disease, suggesting that the imbalance in tau levels in younger individuals might increase the risk of neuronal dysfunction and subsequent neurodegeneration at later stages of the disease. We also demonstrate that tau accumulation in the retina precedes tau build up in the brain. Importantly, even in older mutant mice, which accrue tau in both retinas and brain, the relative increase of tau was consistently higher in retinal than brain samples. These results identify the retina as a highly sensitive system that reflects early tau protein accumulation in AD.

Phosphorylation is a critical post-translational modification of tau during development and in pathological conditions [101]. Inclusions of phosphorylated tau are found in most tauopathies and correlate with severity of disease [73], however, virtually nothing is known about alterations in tau phosphorylation in the AD retina. We found that while tau residues S202 and T205 were highly phosphorylated (AT8), there was a net decrease in the phosphorylation of S199 relative to total tau levels in young mice (PS199). Intriguingly, this pattern was reversed in older animals which displayed increased S199 phosphorylation and decreased phospho-S202/T205, indicative of age-dependent tau modifications at these residues. Although tau hyperphosphorylation has received much attention, accumulating data indicate that oxidative stress, excitotoxicity and starvation induce tau hypophosphorylation [102–104]. Tau dephosphorylation has also been reported during ischemia, hypoxia and glucose deprivation in animal models and in human brain tissue [105–108], indicative of a potential pathological role. Of interest, the alterations in tau phosphorylation observed in 3xTg retinas were different from those we reported in a model of ocular hypertension glaucoma in which S396/S404 residues were hyperphosphorylated, S199 hypophosphorylated, and S202/T205 remained unchanged [4]. Collectively, our observations demonstrate disease-specific changes in retinal tau phosphorylation on key residues.

Tau is an axonal-enriched protein and its abnormal localization to compartments other than the axon, such as soma and dendrites, strongly correlates with neuronal pathology and cognitive decline [109]. RGCs are highly polarized neurons: their soma, dendrites and initial non-myelinated axonal segments are within the retina, whereas the distal myelinated axons are in the optic nerve outside the eye [110]. Our data demonstrate that tau accumulated in RGC dendrites and intraretinal axons, while it was depleted from optic nerve axons in 3xTg mice. This abnormal tau distribution is consistent with pathological changes observed in diseases affecting RGCs such as glaucoma, in which tau accumulates in RGC soma and dendrites leading to neuronal death [4]. Despite the clear redistribution of tau from RGC axons to soma, there was no net increase of tau detected biochemically from 3 to 6 months of age. It is possible that the intracellular redistribution observed in RGCs does not faithfully reflect the global retinal changes detected by western blot analysis. We previously demonstrated that tau is present in retinal cells other than RGCs [4], and tau has also been shown to exist in the extracellular space [111], which could account for this discrepancy. Nonetheless, the pathological properties of tau do not stem only from its accumulation but also from post-translational modifications, most notably phosphorylation. Our data show that the phosphorylation pattern of tau changed dramatically at 6 months relative to younger mice, which might contribute to RGC dysfunction and death.

The lack of changes in tau mRNA levels ruled out transcriptional regulation as a mechanism for tau accumulation in AD retinas. In physiological conditions, tau protein is produced in the cell body and readily sorted to the axon [112], hence the mislocalization of tau in 3xTg RGCs points to the existence of alterations in the sorting mechanisms that control the normal distribution of tau in different neuronal compartments. Changes in tau phosphorylation might reduce its affinity for axonal microtubules and increase it for dendritic microtubules, as shown in cultured spinal cord neurons [113]. Alternatively, retinal tau accumulation might result from impaired degradation in proximal RGC compartments due to defective autophagy or proteasomal pathways and/or altered protein turn over [114, 115]. Future work will be essential to establish the mechanisms driving tau accumulation and missorting in the visual system during the course of AD.

Tau is best known for its role in assembling and stabilizing axonal microtubule networks [116]. In vitro studies have demonstrated that tau can regulate axonal transport primarily by modulating the function of kinesin motor proteins, which mediate anterograde movement [11, 117, 118]. For example, tau overexpression in cultured cells dramatically impairs the anterograde transport of a variety of cargos [119–123]. Previous in vivo studies, however, have yielded controversial results with some reporting reduced anterograde transport in mice overexpressing tau while others showed little or no change [124–126]. Our data using CTβ accumulation in the brain, a readout of active microtubule-dependent transport [88], demonstrate early and substantial deficits in transport along RGC axons in 3xTg mice, which is consistent with previous findings in a model of frontotemporal dementia [47]. Importantly, axonal transport dysfunction was not the result of cell death because transport deficits were detected in young 3xTg mice prior to RGC loss.

To test whether tau accumulation in the retina contributed to axonal transport impairment, we used a siRNA strategy based on the ability to selectively attenuate tau, without completely inhibiting it, and our observation that siRNAs are readily taken up by RGCs when injected into the vitreous space [4, 98]. Although this siRNA approach only partially decreased tau levels in the retina, we observed an improvement of axonal transport in 3xTg RGCs (20%) providing strong proof-of-principle for: 1) a detrimental role of tau accumulation on the regulation of anterograde axonal transport in vivo, and 2) early tau-dependent RGC dysfunction preceding overt neurodegeneration in AD. The decrease of tau burden in the retina appears to have a widespread beneficial effect on the overall health of RGCs leading to improvements in axonal transport and functionality. The mechanism by which pathological tau disrupts anterograde transport in RGCs is currently unknown, but might involve tau detachment and microtubule network destabilization or excessive binding of tau to microtubules resulting in the displacement of kinesin motors [117, 118, 127]. Independent of the mode of action, our findings provide the first demonstration that a global strategy to reduce retinal tau using siRNA is an effective approach to improve axonal transport and attenuate neuronal dysfunction in AD.

## Conclusions

Substantial visual deficits have been documented in Alzheimer’s disease patients; however, the molecular basis of this impairment is poorly understood. This study reveals early and profound alterations in retinal tau including abnormal accumulation, phosphorylation, and missorting. These pathological changes cause substantial retinal neuron dysfunction and subsequent death, suggesting a prominent role for pathological tau in visual defects. The eye is the most accessible part of the CNS and the transparent ocular structures allow swift visualization of the retina. Retinal tau is a promising target to detect early pathological changes and to further understand fundamental mechanisms of neuronal damage in AD and tauopathies.

## Abbreviations

3xTg: PS1(M146 V) APP(Swe) and tau(P301L) triple transgenic mice
AD: Alzheimer’s disease
CNS: central nervous system
CTβ: cholera toxin β subunit
PBS: phosphate buffer saline
qPCR: quantitative polymerase chain reaction
RBPMS: RNA-binding protein with multiple splicing
RGC: retinal ganglion cell
siRNA: short interference RNA
